# Prognostic Factors for Bladder Cancer Patients in Malaysia: A Population-Based Study

**DOI:** 10.3390/ijerph19053029

**Published:** 2022-03-04

**Authors:** Mohd Nasrullah Nik Ab Kadir, Suhaily Mohd Hairon, Najib Majdi Yaacob, Azizah Ab Manan, Nabihah Ali

**Affiliations:** 1 Department of Community Medicine, School of Medical Sciences, Universiti Sains Malaysia, Kubang Kerian 16150, Kelantan, Malaysia; mnasrullah@student.usm.my; 2 Biostatistics and Research Methodology Unit, School of Medical Sciences, Universiti Sains Malaysia, Kubang Kerian 16150, Kelantan, Malaysia; najibmy@usm.my; 3 Timur Laut District Health Office, Penang State Health Department, Ministry of Health Malaysia, Georgetown 11600, Penang, Malaysia; a_drazizah@moh.gov.my; 4 Malaysian National Cancer Registry Department, National Cancer Institute, Ministry of Health Malaysia, Putrajaya 62250, Selangor, Malaysia; nabihah_mm20@iluv.ums.edu.my; 5 Public Health Medicine Department, Faculty of Medicine and Health Sciences, Universiti Malaysia Sabah, Kota Kinabalu 88400, Sabah, Malaysia

**Keywords:** bladder cancer, prognostic factor, population-based, Malaysia

## Abstract

Background: Malaysia has the third highest crude mortality rates of bladder cancer within Southeast Asia. We aimed to identify the prognostic factors for bladder cancer patients in Malaysia. Methods: A retrospective population-based study was conducted among patients diagnosed between 2007 and 2011. Death date until 31 December 2016 was updated. Cox proportional hazard regression analysis was performed to examine clinical variables as prognostic factors of death. Results: Identified prognostic factors of 1828 analyzed patients were age groups, ethnicity, morphology, stage, and surgery. As compared to patients aged 15–44, the adjusted Hazard Ratio for those aged 45–54, 55–64, 65–74, and ≥75 were 1.59, 1.87, 2.46, and 3.47, respectively. Malay and other ethnic groups had 1.22- and 1.40-times the risk of death compared to Chinese. Patients with squamous cell carcinoma were at 1.47-times the hazard of death compared to urothelial carcinoma patients. Stages II, III and IV patients had 2.20-, 2.98-, and 4.12-times the risk of death as compared to stage I. Patients who did not receive surgery were at 50% increased hazard of death. Conclusion: Early detection and/or surgery, especially for those more than 75 years old, Malay, and squamous cell carcinoma could potentially improve survival. The findings could inform national cancer control programs.

## 1. Introduction

Bladder cancer is a malignant neoplasm that originated from any tissues constituting the urinary bladder that frequently occur when the cells are exposed to carcinogens for a prolonged time. Tumors that are confined to the mucosa are known as non-muscle-invasive bladder cancer (NMIBC). Whereas, when the cells invade deeper into the muscle layer, they are called muscle-invasive bladder cancers (MIBC). Bladder cancer is the tenth top cancer worldwide and the ninth leading cancer death among men with an estimated 200,000 deaths in 2018. Among Southeast Asian countries, Singapore is recorded to have the highest incidence of bladder cancer followed by Indonesia, Thailand, and Malaysia with the incidence rate of 2.7 per 100,000 population [1,2,3]. The incidence could be attributed by having a sizable smoking prevalence among adult males (42.4%) in our population [4]. Malaysia is ranked third among regional peers with the highest estimated crude mortality for bladder cancer following Singapore and Thailand [2].

Important clinical determinants influencing bladder cancer prognosis of death are composed of cancer tissue morphology and stage at diagnosis. Non-urothelial bladder cancer specifically squamous cell cancer had the lowest survival [5]. The advanced stage is markedly associated with the highest hazard of death [6,7,8]. Bladder cancer is a disease of old age and is associated with comorbidities. Patients who had multiple comorbidities were at increased risk of mortality [9,10,11]. Bladder cancer treatment consists of surgery, radiotherapy, and chemotherapy. Several articles revealed any treatment receipt at diagnosis could prolong the patient’s life span [12,13,14,15]. Other important sociodemographic factors associated with an increased risk of death were older age [16,17], female sex [18,19,20,21], particular ethnicity [22,23,24], and low socioeconomic status [7,14].

Studies identifying prognostic factors of death for bladder cancer were extensively carried out to understand the disease progression, decide treatment options, and potentially improve survival. However, these studies were mostly conducted in high-income developed countries. The related research topic was lacking in Malaysia, a middle-income country within the southeast Asian region to the best of our knowledge. Our previous work found that the five-year survival was merely 36.9% (95% CI: 34.6, 39.1) and the median survival time was 27.3 months (95% CI: 23.6, 31.0). With adverse characteristics, the survival among our population was markedly lower than the high-income developed countries [25]. With a unique history and culturally diverse ethnicity in Malaysia, reporting local context prognostic factors and the magnitude of each factor associated with all-cause mortality could provide an additional reference for the managing healthcare team to communicate disease trajectory to the patient and caregivers as well as in selecting the appropriate course of action for them. Hence, our analysis aims to identify the prognostic factors of death for bladder cancer patients in Malaysia.

## 2. Materials and Methods

### 2.1. Data Source and Study Design

We retrospectively collected the data of a cohort of bladder cancer patients diagnosed between 1 January 2007 and 31 December 2011 that were passively followed until 31 December 2016 through a population-based cancer surveillance system, the Malaysian National Cancer Registry (MNCR). We described the detailed study design and data source in our previous articles which primarily aimed to describe the overall survival and distribution of the patients’ characteristics [25].

### 2.2. Sample Size Calculation

The sample size was calculated using the two-survival time option for survival analysis in PS: Power and Sample Size Calculation Program version 3.1.6 [26]. Type I error and power of the study were set at 5% and 80%. Drop-outs of 20% were taken into consideration. With median survival time among male patients of 61 months [18], estimated median survival time among female patients of 49 months, the ratio between male to female of 4, accrual time and additional follow-up time of 60 months, the required sample size calculated was 1907. However, the number of bladder cancer cases registered in the MNCR from 2007 to 2011 was 1877 cases [3]. As the total number of cases was fewer than the calculated sample size, all eligible cases were included in the study.

### 2.3. Study Variables

The information provided by the registry was registry number, vital status, death date, date of the last follow-up, age, sex, ethnicity, morphology, stage, surgery, radiotherapy, and chemotherapy received at diagnosis. Age at diagnosis was classified into five groups according to the Malaysian Study on Cancer Survival age grouping [27], which are (i) 15–44, (ii) 45–54, (iii) 55–64, (iv) 65–74, and (v) ≥75 years old. We categorized ethnicity into three major ethnic groups in Malaysia and “others” for an ethnicity other than Malay, Chinese, and Indian. Cancer morphology was divided into four groups based on the International Classification of Diseases for Oncology, Third Edition (ICD-O-3) code. Morphology other than the transitional cell, squamous cell, and adenocarcinoma were grouped as “Others/Nonspecific”.

The cancer stage at diagnosis was based on reporting doctors according to the overall Tumour Node Metastases (TNM) Stage Classification System at diagnosis [3]. Cases without information regarding the stage at diagnosis were classified as “unrecorded”. Surgery treatment received at diagnosis was divided into yes, no, and “unrecorded” for unknown records. According to the data provider, the surgery refers to the definitive surgery. More than half of the radiotherapy and chemotherapy statuses were unrecorded [25], thereby, these variables were not included in our analysis.

### 2.4. Statistical Analysis

We analyzed the data using SPSS version 24. Cox’s proportional hazard regression model was used to identify important prognostic factors. The event for this study was death due to any cause. Patients who were alive on 31 December 2016 or the date of the last follow-up were considered censored. Survival time referred to time calculated in months between incidence date and date of death, or date of study closure or last follow-up date for censored observation. The analyzed variables were age groups, sex, ethnicity, morphology, stage, and surgery received at diagnosis. Variables that were found to have a *p*-value of less than 0.25 in the univariable Cox regression analysis were further evaluated in a multivariable model. Forward and Backward LR methods were used to obtain the preliminary main effect model. All possible two-way interactions were checked, and the proportional hazard assumption was evaluated by hazard functions and log-minus-plot. The final model was presented as adjusted Hazard Ratio (Adj. HR), 95% confidence intervals (CI), Wald statistic, and p-value. All p-values were two-sided, and *p* < 0.05 were considered statistically significant.

### 2.5. Ethics Statement

Ethical clearances were obtained from the Human Research and Ethics Committee, Universiti Sains Malaysia (USM/JEPeM/18100500) and the Medical Research and Ethics Committee, Ministry of Health Malaysia (NMRR-18-2965-44397 (IIR)). We obtained permission to use the non-identifying patient records from the data custodian, the Directors of the National Cancer Institute, Ministry of Health, Putrajaya. Confidentiality of the patients’ information was well looked after, as only researchers had access to the data. These data were used under agreement for the current study and are not publicly available without the explicit permission of the Director General, Ministry of Health Malaysia.

## 3. Results

### 3.1. Patients’ Profiles

More than half (67.6%) of 1828 patients died at the end of the study period. Patients’ profiles according to variable categorization and last follow-up status are presented in Table 1.

### 3.2. Cox Regression Analysis

By univariable Cox regression analysis, prognostic factors with a p-value of less than 0.25 identified were age groups, ethnicity, morphology, stage, and surgery at diagnosis as displayed in Table 2. These variables were further analyzed with multivariable Cox regression. We excluded sex variables in the multivariable analysis. All five variables were found to be significant and included in the final model after employing the forward and backward LR variable selection method. All possible two-way interactions were checked and found to be insignificant. The final model met the proportional hazard assumption in Cox regression analysis based on hazard function and log-minus-log plot.

Results of the multivariable Cox regression analysis revealed patients aged 45–54, 55–64, 65–74, and ≥75 years old had 1.6-, 1.9-, 2.5-, and 3.5-times hazard of death, respectively, as compared to patients aged 15-44 years old. Malay and other ethnic group bladder cancer patients were at 22% and 40% increased hazard of death compared to Chinese. Squamous cell carcinoma patients had 1.5-times the hazard of mortality compared to urothelial carcinoma. As compared to stage I patients, stages II, III, and IV patients had 2-, 3-, and 4-times hazard of death. Patients who did not receive surgery at diagnosis were 1.5-times at risk of death compared to those who had surgery. The detailed result of the analysis is presented in Table 3.

## 4. Discussion

We conducted a study to assess prognostic factors of death among bladder cancer patients diagnosed between 2007 and 2011 using a population-based cancer database. We found specific age groups, ethnicity, morphology, stage, and surgery at diagnosis were significantly associated with death. However, our result showed no significant difference in mortality risk between men and women. Other studies mentioned that the possible reasons include a similar stage at presentation and morphology between both genders [7,8,10,14,16,22]. In contrast, a meta-analysis study involving 30,039 patients who had radical cystectomy revealed that female patients were at higher risk of death (HR 1.08, 95% CI: 1.03, 1.12) compared to men [28].

Ethnicity is one of the key social determinants of health that contribute to discrepancies in health outcomes, especially among the disadvantaged groups. In the United States, African Americans were associated with poor prognosis compared to Caucasian Americans [12,14,20,23]. Malaysia is composed of three major ethnic groups with a distinct history, culture, and socioeconomic status. Malay ethnic group which is generally associated with lower socioeconomic level was also reported to have lower survival compared to Chinese and Indian in other common cancer such as female breast, colorectal, and lung cancer [27]. Breast and cervical cancer studies in Malaysia suggested the late-stage presentation among Malay as the key contributing factor to the increased hazard of death. The late-stage presentation could be due to culturally influenced factors such as health-seeking behavior, health belief, and financial resources. Malay and other minority ethnic groups tend to seek alternative therapy and are not complied with long-term multimodal serial intervention and cancer surveillance follow-up [29,30,31].

Our study showed that increased age was significantly associated with a higher hazard of death. Similarly, a study in Japan revealed patients aged ≥75 and 65–74 years had 2.5- and 1.4-times the risk of death compared to those aged <65 years [21]. Similar trends were reported by other studies in Canada, the Netherlands, and the United States as elderly patients are associated with multiple comorbidities which hinder them from receiving standard treatment such as radical cystectomy among muscle-invasive bladder cancer patients or systemic chemotherapy among metastatic diseases [8,9,14,20,21].

Regarding tissue morphology, the increased hazard of death among squamous cell carcinoma patients in our study agreed with other numerous studies. Patients diagnosed with the morphology were at almost two times the risk of mortality compared to urothelial carcinoma patients [5]. They were less likely to be diagnosed at an early stage and associated with muscle invasion, thereby leading to poor prognosis [5,6,11,22,32].

The cancer stage describes the extent of cancer spread and represents the severity of the disease at presentation. A higher stage is associated with an increased hazard of death as found in our study and multiple bladder cancer prognostic studies [6,9,23]. A study using Surveillance, Epidemiology, and End Results (SEER) population-based data revealed stages II, III and IV bladder cancer patients had 3-, 3.5-, and 8-times hazard of death compared to stage I patients [6]. These studies further highlighted the importance of early detection. Patients that present with blood in the urine should be further evaluated by cystoscopy examination. As for the time being, there is no recommendation for mass population screening of bladder cancer [1,33].

Surgery is the primary treatment for non-metastatic bladder cancer. Surgical treatment for NMIBC is an endoscopic transurethral resection of bladder tumour (TURBT), and for MIBC is radical cystectomy [1]. Generally, bladder cancer patients in Malaysia who did not undergo definitive surgery (which could be either TURBT or radical cystectomy) were at an increased hazard of death compared to those who had surgery. Our results correspond to other studies in Australia [7], the Netherlands [9], and the United States [7,20,22]. Recognizing the importance of surgical treatment, inadequate specialized urological services nationwide should be expanded to improve accessibility, minimize treatment delay, and refusal [34].

The first limitation of our study was the omission of variables such as socioeconomic status, comorbidity status, radiotherapy, and chemotherapy status. These variables were not available or incomplete in the MNCR database. Secondly, surgical treatment information was limited and without an exact type of surgery (TURBT or radical cystectomy). Surgical treatment variables were only provided at initial diagnosis. The subsequent intervention was not available in the database that could influence patients’ prognosis. Thirdly, this study is a retrospective review using previously available records. The study design may introduce biases in the analysis compared to the prospective research method.

The strength of our study is that it was population-based, and that the death dates were updated with the national mortality register ensuring completeness of death status. As far as we know, this is the largest sample using recently available data covering nationwide bladder cancer patients despite the limitations of our cancer surveillance system. The findings could reflect the overall bladder cancer care status in Malaysia. Future studies should include the mentioned factors across clinical and social determinants of health to better address the prognostic factors of death for bladder cancer patients in Malaysia.

## 5. Conclusions

In summary, increasing age, Malay ethnicity, squamous cell carcinoma morphology, late-stage, and patients who did not undergo surgery had a poor prognosis. Early detection and surgical intervention among these patients might improve survival. The prognostic factors which had been identified would provide an additional reference in strengthening the prevention and control programs for bladder cancer patients in Malaysia.

## Figures and Tables

**Table 1:** Profile of bladder cancer patients included in the study (*n* = 1828).

Variables	*n*	Last Follow-Up Status, *n* (%)	
		Alive = Censored	Dead = Event
All patients	1828	593 (32.4)	1235 (67.6)
Age groups (years)			
15–44	118	72 (61.0)	46 (39.0)
45–54	236	103 (43.6)	133 (56.4)
55–64	463	178 (38.4)	285 (61.6)
65–74	603	175 (29.0)	428 (71.0)
≥75	408	65 (15.9)	343 (84.1)
Sex			
Male	1438	455 (31.6)	983 (68.4)
Female	390	138 (35.4)	252 (64.6)
Ethnicity			
Malay	903	250 (27.7)	653 (72.3)
Chinese	684	247 (36.1)	437 (63.9)
Indian	107	45 (42.1)	62 (57.9)
Others	134	51 (38.1)	83 (61.9)
Morphology			
Urothelial carcinoma	1430	488 (34.1)	942 (65.9)
Squamous cell carcinoma	57	12 (21.1)	45 (78.9)
Adenocarcinoma	150	44 (29.3)	106 (70.7)
Others/Nonspecific	191	49 (25.7)	142 (74.3)
Stage			
I	270	162 (60.0)	108 (40.0)
II	216	59 (27.3)	157 (72.7)
III	153	27 (17.6)	126 (82.4)
IV	250	32 (12.8)	218 (87.2)
Unrecorded	939	313 (33.3)	626 (66.7)
Surgery			
Yes	902	344 (38.1)	558 (61.9)
No	297	64 (21.5)	233 (78.5)
Unrecorded	629	185 (29.4)	444 (70.6)

**Table 2:** Prognostic factors of death by univariable Cox proportional hazard regression (*n* = 1828).

Variables	*b*	Crude HR (95% CI)	Wald Statistic	*p*-Value
Age groups (years)				
15–44	0	1		
45–54	0.52	1.69 (1.21, 2.36)	9.33	0.002
55–64	0.65	1.92 (1.41, 2.63)	16.96	<0.001
65–74	0.89	2.43 (1.79, 3.29)	32.53	<0.001
≥75	1.27	3.57 (2.62, 4.86)	65.29	<0.001
Sex				
Male	0	1		
Female	0.03	1.03 (0.89, 1.18)	0.13	0.721
Ethnicity				
Malay	0	1		
Chinese	0.27	1.31 (1.16, 1.47)	18.65	<0.001
Indian	−0.11	0.90 (0.69, 1.17)	0.65	0.418
Others	0.24	1.27 (1.00, 1.61)	3.98	0.046
Morphology				
Urothelial carcinoma	0	1		
Squamous cell carcinoma	0.54	1.71 (1.27, 2.31)	12.35	<0.001
Adenocarcinoma	0.18	1.19 (0.98, 1.46)	2.95	0.086
Others/Nonspecific	0.36	1.43 (1.20, 1.71)	16.06	<0.001
Stage				
I	0	1		
II	0.91	2.50 (1.95, 3.19)	53.37	<0.001
III	1.19	3.28 (2.54, 4.25)	81.61	<0.001
IV	1.62	5.03 (3.99, 6.35)	184.7	<0.001
Unrecorded	0.89	2.43 (1.98, 2.99)	72.64	<0.001
Surgery				
Yes	0	1		
No	0.55	1.73 (1.48, 2.01)	48.74	<0.001
Unrecorded	0.36	1.43 (1.27, 1.62)	31.99	<0.001

*b*, crude regression coefficient; HR, Hazard Ratio; CI, Confidence Interval.

**Table 3:** Prognostic factors of death by multivariable Cox proportional hazard regression (*n* = 1828).

Variables	*n* (%)	Adjusted HR (95% CI)	Wald Statistic	*p*-Value
Age groups (years)				
15–44	118 (6.5)	1		
45–54	236 (12.9)	1.59 (1.14, 2.23)	7.26	0.007
55–64	463 (25.3)	1.87 (1.37, 2.56)	15.26	<0.001
65–74	603 (33.0)	2.46 (1.81, 3.34)	33.31	<0.001
≥75	408 (22.3)	3.47 (2.54, 4.73)	61.71	<0.001
Ethnicity				
Malay	903 (49.4)	1		
Chinese	684 (37.4)	1.22 (1.08, 1.38)	9.8	0.002
Indian	107 (5.9)	0.91 (0.69, 1.18)	0.53	0.466
Others	134 (7.3)	1.40 (1.10, 1.79)	7.59	0.006
Morphology				
Urothelial carcinoma	1430 (78.2)	1		
Squamous cell carcinoma	57 (3.1)	1.47 (1.09, 1.99)	6.24	0.012
Adenocarcinoma	150 (8.2)	1.12 (0.92, 1.37)	1.22	0.269
Others/Nonspecific	191 (10.5)	1.16 (0.97, 1.40)	2.72	0.099
Stage				
I	270 (14.8)	1		
II	216 (11.8)	2.20 (1.71, 2.82)	38.69	<0.001
III	153 (8.4)	2.98 (2.30, 3.87)	67.93	<0.001
IV	250 (13.7)	4.12 (3.24, 5.25)	133.12	<0.001
Unrecorded	939 (51.4)	2.02 (1.63, 2.49)	41.96	<0.001
Surgery				
Yes	902 (49.3)	1		
No	297 (16.3)	1.46 (1.25, 1.71)	22.75	<0.001
Unrecorded	629 (34.4)	1.22 (1.07, 1.39)	9.06	0.003

HR, Hazard Ratio; CI, Confidence Interval; no significant two-way interaction; proportional hazard assumption was met as evaluated by hazard function plot and log-minus-log plot.

## Data Availability

The data that support the findings are available from the National Cancer Institute, Ministry of Health Malaysia, but restrictions apply to the availability of these data. These data were used under agreement for the current study and are not publicly available. Data are, however, available from the authors but only with the explicit permission of the Director-General, Ministry of Health Malaysia.
